# Artificial intelligence fusion for predicting survival of rectal cancer patients using immunohistochemical expression of Ras homolog family member B in biopsy

**DOI:** 10.37349/etat.2023.00119

**Published:** 2023-02-07

**Authors:** Tuan D. Pham, Vinayakumar Ravi, Bin Luo, Chuanwen Fan, Xiao-Feng Sun

**Affiliations:** 1 Center for Artificial Intelligence, Prince Mohammad Bin Fahd University, Khobar 34754, Saudi Arabia; 2 Department of Biomedical and Clinical Sciences, Linköping University, 58183 Linköping, Sweden; 3 Department of Gastrointestinal Surgery, Sichuan Provincial People’s Hospital, Chengdu 610032, Sichuan, China; University of Campania “L. Vanvitelli”, Italy

**Keywords:** Artificial intelligence, biomarkers, immunohistochemistry, machine learning, precision medicine, proteins, rectal neoplasms

## Abstract

**Aim:**

The process of biomarker discovery is being accelerated with the application of artificial intelligence (AI), including machine learning. Biomarkers of diseases are useful because they are indicators of pathogenesis or measures of responses to therapeutic treatments, and therefore, play a key role in new drug development. Proteins are among the candidates for biomarkers of rectal cancer, which need to be explored using state-of-the-art AI to be utilized for prediction, prognosis, and therapeutic treatment. This paper aims to investigate the predictive power of Ras homolog family member B (RhoB) protein in rectal cancer.

**Methods:**

This study introduces the integration of pretrained convolutional neural networks and support vector machines (SVMs) for classifying biopsy samples of immunohistochemical expression of protein RhoB in rectal-cancer patients to validate its biologic measure in biopsy. Features of the immunohistochemical expression images were extracted by the pretrained networks and used for binary classification by the SVMs into two groups of less and more than 5-year survival rates.

**Results:**

The fusion of neural search architecture network (NASNet)-Large for deep-layer feature extraction and classifier using SVMs provided the best average classification performance with a total accuracy = 85%, prediction of survival rate of more than 5 years = 90%, and prediction of survival rate of less than 5 years = 75%.

**Conclusions:**

The finding obtained from the use of AI reported in this study suggest that RhoB expression on rectal-cancer biopsy can be potentially used as a biomarker for predicting survival outcomes in rectal-cancer patients, which can be informative for clinical decision making if the patient would be recommended for preoperative therapy.

## Introduction

The Precision (PRE) Medicine Initiative announced in 2015 is a new medical research effort to replace the practice of “one-size-fits-all” with patient-specific treatment in health care [1]. A key success of PRE medicine is the discovery of disease biomarkers for the prevention, prediction, prognosis, and treatment of diseases [2, 3]. This initiative has been found to be promising in clinical trials and its success is evidenced by increasing new drug approvals [4]. While good efforts for obtaining the whole molecular information of patients are being pursued in clinics, however, only a small number of molecular biomarkers have been used for targeted drug development in current PRE medicine. Artificial intelligence (AI) methods are realized as the cutting-edge technology for enabling to comprehend the patients’ heterogeneous landscapes across their molecular profiles so that new drugs can be made for personalized prevention and treatment [4].

In colorectal cancer (CRC), ensemble classification and regression were sequentially used as machine-learning methods for predicting the survival chance and time of patients with CRC, respectively [5]. An application of AI in CRC used 10 pretrained convolutional neural networks (CNNs) for classifying N-terminally truncated p73 (DNp73) expression identified by immunohistochemistry (IHC) on the biopsy and surgically resected tumor samples obtained from a cohort of rectal cancer patients [6]. Average validation results reported in the study showed high accuracy (ACC) rates for the prediction of less or more than 5-year survival rate of the rectal-cancer patients either with or without preoperative radiotherapy (pRT). Such a finding can be helpful for clinical decision making if the patient would be treated with pRT based on the predicted survival outcome [6].

Another AI-based diagnosis system, which applied deep learning for pattern classification, was developed for detecting patients with CRC using both quantitative and qualitative data, including pathology [7]. As in another study, 10 CNNs were trained for classifying images of heterogeneous hematoxylin and eosin (HE) stained tumor tissues that were obtained from four cohorts of CRC patients with good or poor disease outcomes [8]. The study discovered a prognostic marker that can locate stage II and III patients with CRC into separate groups. Such use of AI in HE image screening opens a new door to assisting clinicians in preventing low-risk cohorts from having unnecessary therapy, and otherwise recommending appropriate types of treatment that would produce good effects on other groups of patients. Similarly, AI-based classification of histopathology images was applied for detecting and diagnosing CRC patients [9]. Not only the AI tool could much more accurately perform the tasks than being done by pathologists but also was able to significantly alleviate the conventional workload that causes fatigue to human-based assessment [9]. Another study applied machine learning of clinical and next-generation sequencing data for identifying molecular characteristics in patients with metastatic CRC treated with oxaliplatin-based chemotherapy [10]. The identified biomarker can assist in clinical decision making if patients with advanced CRC should have alternative regimens to adjuvant therapy, where the latter would not benefit the patients [10].

Using whole slide images, a deep-learning approach was developed for predicting the survival time of patients with CRC in stages II and III [11]. This study suggested new prognostic features that need to be further validated in future clinical trials. Novel applications of AI in CRC have been recently reviewed and discussed how the technology would help to reduce the risk and occurrence of cancer and lead to personalized treatment of the disease by means of its promising ability to discover patterns and causes of CRC [12].

Here the power of Ras homolog family member B (RhoB) protein as a biomarker in rectal cancer biopsy is examined from the standpoint of AI and machine learning. RhoB is a member of the Rho family of proteins, whose function takes part in the regulation of the actin cytoskeleton, cell adhesion, and cell growth [13]. Evidence of tumor formation was found in mice when RhoB was deleted [14]. Another study indicated the correlation of RhoB expression in clear cell renal cell carcinoma (ccRCC) carcinogenesis and progression [15]. These findings strongly suggest that RhoB is one of the potential targets for anti-cancer therapeutics [13, 15].

A study found that RhoB expression plays a critical role in the radioresistance of CRC through Ak strain transforming (Akt) and forkhead box protein M1 (FOXM1) pathways [16]. However, our manual pathologist-based analysis could not confirm the predictive value of RhoB expression in biopsy samples of CRC patients. A recent investigation has reported the use of tensor decomposition of convolutional eigenvalues of RhoB expression in IHC images of biopsy collected from two groups of rectal-cancer patients who had survival rates of less or more than 5 years [17]. By using all IHC biopsy samples, the method of tensor decomposition could differentiate between the factor distributions of the two groups of patients without pRT, who had different survival rates, but the study did not carry out the cross-validation (classification) of the samples.

This study introduces the combination of pretrained CNNs and a support vector machine (SVM) algorithm for discovering the power of RhoB expression on IHC-stained biopsy samples obtained from two cohorts of rectal-cancer patients having less or more than 5 years of survival after surgery.

## Materials and methods

### Patients’ data

This study included rectal cancer patients from the southeast health care region of Sweden between 1984 and 2013. About half of the patients received both pRT and surgery, and the rest had surgery alone. All patients were followed up to more than five years or death. Biopsy samples were obtained from the primary tumor of the patients before radiotherapy (RT) or surgery, fixed with formalin, embedded in paraffin, and made as tissue microarray (TMA). The TMAs were sliced into 4 μm sections and treated slides following the immunohistochemical procedures. The slides were incubated with primary antibody (anti-RhoB mouse monoclonal, sc-8048), secondary antibody, and envision system labelled polymer-HRP anti-mouse. For visualization, diaminobenzidine (DAB) was applied to the slides followed by counterstaining with HE staining solution and mounted with Pertex mounting media.

Biopsy samples included in this study were taken from 53 patients without pRT from the randomized Swedish rectal cancer trial of pRT [18], whose clinical and pathological characteristics are described in Table 1. The image set consists of 120 IHC-stained biopsy samples (mean of 2.26 samples per patient), of which 45 images are of survival rate of < 5 years, and 75 images are of > 5 years. The ratio of < 5-year image set to > 5-year image set (45:75) indicates a data imbalance in the two class labels, which usually happens in many medical datasets. The 10-fold cross-validation, which is a method for evaluating predictive models by randomly splitting 90% of the original dataset into a training set to train the model, and 10% of the original dataset into a test set to evaluate its performance, was used to compute various performance measures of the classification models. For this binary classification of IHC images of RhoB expression in rectal cancer biopsy (either < or > 5 years of survival), > 5-year survival is considered as positive (P), and < 5-year as negative (N). The numbers of samples of > and < 5 years of survival are denoted as P and N, respectively. True P (TP) is the number of samples of > 5 years that are correctly classified as > 5 years, whereas true N (TN) is the number of samples of < 5 years being correctly classified as < 5 years. False P (FP) is the number of samples of < 5 years that are misclassified as > 5 years. False N (FN) is the number of samples of > 5 years that are misclassified as < 5 years. Formulae for calculating classification ACC, sensitivity (SEN) or TP rate, specificity (SPE) or TN rate, PRE or P predictive value, and F_1_ score are given in Table 2.

**Table 1.** Clinical and pathological characteristics of included rectal cancer patients

**Characteristics**	**Surviving > five years (%) *n* = 33**	**Surviving < five years (%) *n* = 20**	**Chi-squared test**	***P*-value**
Sex			0.37	0.54
Male	19 (57)	9 (45)		
Female	14 (43)	11 (55)		
Age (years)			0.55	0.46
< 70	15 (45)	12 (60)		
≥ 70	18 (55)	8 (40)		
Tumor location (cm)	6 (2–14)	4 (1–17)	198.5	< 0.05
Operation type			0.45	0.5
Anterior resection	16 (48)	7 (35)		
Abdominoperineal resection	17 (52)	13 (65)		
pTNM stage			*	< 0.05
I	14 (42)	0 (0)		
II	13 (39)	2 (10)		
III	6 (18)	15 (75)		
IV	0 (0)	3 (15)		
Local recurrence			7.28	< 0.05
No	31 (94)	12 (60)		
Yes	2 (6)	8 (40)		
Distal recurrence			21.6	< 0.05
No	29 (88)	4 (20)		
Yes	4 (12)	16 (80)		

*: Fisher’s exact test; pTNM: pathological tumor, node, and metastasis

**Table 2.** Performance measures of classification models

**ACC**	**SEN**	**SPE**	**PRE**	**F_1_**
$\frac{TP\;+\;TN}{P\;+\;N}$	$\frac{TP}{P}$	$\frac{TN}{N}$	$\frac{TP}{TP\;+\;FP}$	$\frac{2TP}{2TP\;+\;FP\;+\;FN}$

### CNN-based feature extraction of IHC images

Pretrained CNNs for image classification have been trained to extract differentiable features from natural images of various classes. These pretrained deep networks can be used for learning and recognizing new classes. Most of these pretrained networks were trained on subsets of the ImageNet database 20, which include more than a million images of 1,000 different categories. CNN architectures consist of many convolutional layers. While early layers at the beginning of the networks have a small receptive field size to learn low-level or simple features, deeper network layers at the end of the networks have larger receptive field sizes to learn high-level or complex features.

The three main layers of a CNN are convolutional, pooling, and fully connected layers. The basic purpose of the pooling layer is to down-sample the feature map learned by the convolutional layer. As a result of feature compression, it can help generalize the features to avoid overfitting in the model training. In average pooling, the layer computes the average values of elements in the feature map, resulting in producing the average down-sampled feature map that can be translation-invariant. Furthermore, the use of the global average pooling offers an advantage over the fully connected layers in that its structure is closer to the convolution layer that contains a set of filters for building the deep feature map of an input image [19]. This study adopted 6 pretrained CNNs for the deep feature extraction of the IHC images of RhoB expression on the biopsy samples of the two groups of rectal-cancer patients whose survival rates were < and > 5 years, respectively. These 6 CNNs, which were pretrained using the ImageNet database [20], represent a spectrum of publicly available networks of various sizes, layers, and parameters. The basic properties of these pretrained CNNs are shown in Table 3. These networks and their layers used for feature extraction of the IHC images are briefly described herein because the 6 pretrained CNNs used in this study are very well documented in the literature. The interested reader is referred to the cited references for detailed descriptions of the 6 pretrained deep-learning models.

**Table 3.** Properties of pretrained networks

**Pretrained CNN model**	**Layers**	**Model size (MB)**	**Parameters**	**Input size (pixels)**
ResNet-18	18	44	11.7 × 10^6^	224 × 224
SqueezeNet	18	5.2	1.24 × 10^6^	227 × 227
DenseNet-201	201	77	20 × 10^6^	224 × 224
AlexNet	8	227	61 × 10^6^	227 × 227
Xception	71	85	22.9 × 10^6^	299 × 299
NASNet-Large	1244^#^	332	88.9 × 10^6^	331 × 331

# indicates the network does not consist of a linear sequence of modules; NASNet: neural search architecture network; ResNet: residual network; DenseNet: dense convolutional network

(1) ResNet-18: This net is a version of a pretrained CNN called ResNet, which consists of 18 layers [21]. ResNet introduced the notion of the identity shortcut connection that stacks layers (skipping non-essential layers) on current networks that are still capable of providing the same performance [21]. In this study, layer “pool5” (global average pooling features = 512) was selected for feature extraction of the IHC images.
(2) SqueezeNet: It is a pretrained CNN, which is 18 layers deep [22, 23]. With the use of fire modules, which consist of “squeeze” convolution layers having only 1 × 1 filters, the net was created to have fewer parameters for a neural network so that it can be easily implemented on computers of standard memory and transmitted over computer networks. In this study, layer “pool0” (global average pooling features = 1000) was selected for feature extraction of the IHC images.
(3) DenseNet-201: This pretrained CNN is a variant of the DenseNet [24]. DenseNet-201 has 201 layers, where subsequent layers receive inputs from the previous layers by concatenation. Such an architecture that allows collective information received from the prior layers can reduce the number of channels, making the network become dense. In this study, layer “avg_pool” (global average pooling features = 1920) was selected for feature extraction of the IHC images.
(4) AlexNet: It is 8 layers deep [25], where the first 5 layers are convolutional, some followed by max-pooling layers, and the last 3 layers are fully connected. By using the non-saturating rectified linear activation function, the network was shown to be able to improve its training performance over tanh and logistic sigmoid activation functions [25]. In this study, layer “pool15” (max-pooling features = 256, whereas global average pooling features do not exist) was selected for feature extraction of the IHC images.
(5) Xception: The term Xception refers to extreme inception that is of depthwise separable convolutions [26]. This pretrained CNN consists of 71 layers. The network was designed with repeating modules called inceptions, where each of which carries out different transformations of the same input. Multiple transformed results obtained from the inception modules are combined and selected by the network for deep learning. In this study, layer “avg_pool” (global average pooling features = 2048) was selected for feature extraction of the IHC images.
(6) NASNet-Large: This is a variant of the NASNet models [27]. This net was designed to consist of normal and reduction cells to carry out search space, search strategy, and performance estimation to identify the best algorithm in order to achieve the best optimal performance over a certain piece of work. The NASNet search requires a large amount of computing processing for searching and training with particular reference to computer vision problems to achieve the best results [28]. In this study, layer “global_average_pooling2d_2” (global average pooling features = 4032) was selected for feature extraction of the IHC images.

### SVM-based classification of IHC images

As pretrained CNNs can be utilized for obtaining effective features of images of new classes by using their layer activations. These activation-based features can be readily adopted for training an SVM for binary image classification. The rationale for extracting learned image features from a pretrained CNN and then using these features for training an SVM is that not only the extraction of pretrained CNN-based features is computationally efficient but also has been proven more effective for binary classification of pathological images by an SVM classifier than the transfer learning [29, 30]. Particularly when the new dataset is relatively very small, it can be anticipated that the fine-tuning of pretrained deeper layers cannot improve the classification performance as there is little information to learn from the new data.

The SVM classifier tries to find a hyperplane that can well separate class features into two different groups for pattern classification. The classifier used in this study for learning the pretrained CNN-based features of the IHC images is a linear SVM binary learner. The linear SVM classifier works as follows [31, 32]. Let *x* = *x*_1_, …, *x_N_* be the set of CNN-based feature vectors of *N* IHC images. Each *x_i_*, *i* = 1, …, *N*, is classified as class *y_i_* = 1 for the survival of > 5 years (P class) or *y_i_* = –1 for the survival of < 5 years (N class). To determine a hyperplane for classification, the linear SVM function, which classifies feature set *x*, is defined as:

(1) $$f(x)=w^{T}x+b=\sum_{i\;=\;1}^{N}w_{i}x_{i}+b=0,$$

where two parameters *w* and *b*, which are used to identify the hyperplane, are the vector of coefficients and a bias (scalar), respectively.

The two hyperplanes can be determined as *f*(*x_i_*) = 1 if *y_i_* = 1 and *f*(*x_i_*) = −1 if *y_i_* = –1. These conditions alternatively yield *y_i_f*(*x_i_*) = *y_i_*(*w^T^x_i_* + *b*) ≥ 1, *i* = 1, …, *N*. The optimal margin length is $\frac{1}{2}\left\|w\right\|$, giving the optimization problem

(2) $$\begin{array}{l} \text{Minimize}\;\frac{1}{2}{\left\|w\right\|}^{2}\\ \text{subject}\;\text{to}\;y_{i}(w^{T}x_{i}+b)\geq 1,i=1,\ldots ,N. \end{array}$$

The objective function expressed in Eq. (2) can be optimized using the method of Lagrange multipliers, which introduces coefficients *α*_1_, …, *α_N_* as:

(3) $$\begin{array}{l} \text{Minimize}\;L(\alpha )=\frac{1}{2}\sum_{i,j=1}^{N}\alpha_{i}\alpha_{j}y_{i}y_{j}x_{i}^{T}x_{j}-\sum_{i=1}^{N}\alpha_{i}\\ \text{subject}\;\text{to}\;\sum_{i=1}^{N}\alpha_{i}y_{i}=0;\alpha_{i}\geq 0,i=1,\ldots ,N. \end{array}$$

By solving Eq. (3) for *α_i_*, *i* = 1, …, *N*, the resulting linear SVM algorithm that classifies feature set *x* by using the following sign function as:

(4) $$f(x)=sign\left[\sum_{i,\;j\;=\;1}^{N}\alpha_{i}y_{i}x_{i}^{T}x_{j}+b\right].$$

**Figure 1.**
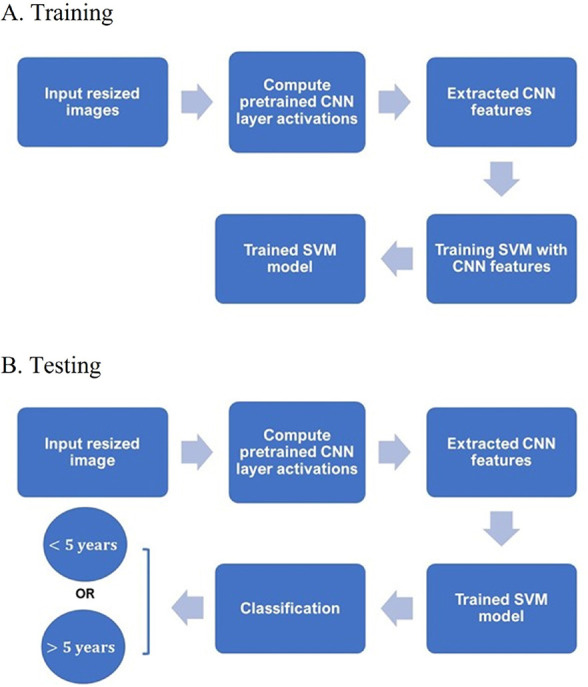
Training and testing procedures. A. Diagram of main steps for building an SVM classifier with extracted CNN features of training IHC and B. testing the trained SVM classifier in predicting the survival time given a test IHC

A diagram showing the main steps for classifying the IHC images into either < 5-year or > 5-year class using CNN and SVM is given in Figure 1.

## Results

For the direct classification of IHC images using ResNet-18, SqueezeNet, DenseNet, AlexNet, Xception, and NASNet-Large, parameters for the transfer learning of the pretrained deep-learning models were specified as: minimum batch size = 30, the maximum number of epochs = 20 (convergence reached), initial learning rate = 0.0003, number of epochs for dropping the learning rate = 10, factor for dropping the learning rate = 0.1, factor for L_2_ regularization (weight decay) = 0.0001, gradient threshold method = L_2_ norm, gradient threshold = ∞, and training and validation data were shuffled once before training using stochastic gradient descent with momentum.

The average results of the statistical measures (see Table 4) obtained from 5 runs of 10-fold cross-validation, which were obtained from 6 individual pretrained CNNs (ResNet-18, SqueezeNet, DenseNet, AlexNet, Xception, and NASNet-Large), and the extracted features of these pretrained CNNs for classification with the SVM (ResNet-18 & CNN, SqueezeNet & CNN, DenseNet & CNN, AlexNet & CNN, Xception & CNN, and NASNet-Large & CNN). The *P*-values of all the statistical measures are less than 0.00001.

**Table 4.** Ten-fold cross-validation results of binary classification of RhoB expression in rectal-cancer biopsy

**Methods**	**ACC (%)**	**SEN (%)**	**SPE (%)**	**PRE (%)**	**F_1_**
ResNet-18	59.09 ± 8.83	75.71 ± 15.13	30.00 ± 19.72	65.65 ± 5.86	0.70 ± 0.08
ResNet-18 & SVM	67.28 ± 10.36	68.57 ± 27.48	65.00 ± 28.50	81.43 ± 12.21	0.70 ± 0.16
SqueezeNet	61.82 ± 10.32	70.00 ± 22.79	47.50 ± 21.89	71.75 ± 11.12	0.68 ± 0.14
SqueezeNet & SVM	67.28 ± 4.98	77.14 ± 21.30	50.00 ± 46.77	80.72 ± 17.78	0.73 ± 0.12
DenseNet-201	60.00 ± 8.78	72.86 ± 10.54	37.50 ± 24.30	68.11 ± 8.91	0.70 ± 0.07
DenseNet-201 & SVM	69.09 ± 10.36	82.86 ± 18.63	45.00 ± 20.92	78.89 ± 7.40	0.77 ± 0.10
AlexNet	54.55 ± 17.14	74.29 ± 25.02	20.00 ± 19.72	60.34 ± 14.70	0.66 ± 0.18
AlexNet & SVM	72.74 ± 11.13	85.71 ± 0.00	50.00 ± 30.62	76.67 ± 13.69	0.80 ± 0.07
Xception	60.91 ± 9.63	80.00 ± 13.36	27.50 ± 34.26	68.48 ± 13.20	0.72 ± 0.06
Xception & SVM	74.55 ± 7.61	74.29 ± 18.63	75.00 ± 30.62	87.14 ± 13.01	0.78 ± 0.08
NASNet-Large	71.82 ± 15.72	77.14 ± 22.54	62.50 ± 21.25	78.28 ± 11.94	0.76 ± 0.15
NASNet-Large & SVM	84.55 ± 7.48	90.00 ± 11.76	75.00 ± 20.41	87.70 ± 9.55	0.88 ± 0.06

The combination of NASNet-Large & SVM achieved the best average values with ACC = 85%, SEN = 90%, SPE = 75%, PRE = 88%, and F_1_ score = 0.9. The results particularly show the robust prediction of biopsy samples of > 5-year survival rate (90%). Without the use of the SVM, NASNet-Large provided ACC = 72%, SEN = 77%, SPE = 63%, PRE = 88%, and F_1_ = 0.8, which are lower than those obtained by the NASNet-Large & SVM. The second highest ACC (75%) was obtained from the Xception & SVM that also resulted in SEN = 74%, SPE = 75%, PRE = 87%, and F_1_ = 0.78. The performance ranking of the other 4 individual pretrained CNN classifiers from highest to lowest ACC rates are: SqueezeNet (62%), DenseNet-201 (60%), ResNet-18 (59%), and AlexNet (55%). The performance ranking of the other 4 classifiers with the combination of the pretrained CNNs & SVM from highest to lowest ACC rates are: AlexNet & SVM (73%), DenseNet-201 & SVM (69%), and either ResNet-18 & SVM (67%) or SqueezeNet & SVM (67%).

## Discussion

The high statistical performance measures provided by the NASNet-Large & SVM strongly suggest the potential of RhoB expression captured on the IHC samples of the rectal-cancer biopsy as a protein biomarker for predicting the 5-year survival rate of rectal-cancer patients without undergoing pRT. Such finding of the discrimination power of RhoB in rectal-cancer biopsies is useful for clinical decision making and can be of guidance for recommending treatments to rectal-cancer patients. It has been known that invasive surgical operations for patients with cancer can be prevented if their short-term survival expectancy after diagnosis can be correctly predicted, and treatment can be provided to cancer patients with longer time of survival expectancy [33]. This kind of pathological prediction can only be carried out using biopsies. In fact, the molecular information of tumors has been increasingly utilized for predicting response to cancer treatments, and the use of image-guided needle biopsy and biopsy-based biomarkers play an essential role in PRE medicine that promises a path to accurate prognosis, prediction of treatment response, or resistance in cancers [33, 34]. The use of advanced AI and machine learning for discovering intricate and complex patterns in tumor biopsies can open new doors to assisting clinical researchers in studying the effects and pharmacodynamics of cancer treatment drugs and timely identification of relevant biomarkers [35, 36].

The combination of pretrained CNNs (used for feature extraction) and SVM (used for pattern classification) appears to be a useful machine-learning tool for discovering or validating biomarker candidates through their molecular expression on biopsies, such as RhoB expression on IHC imaging as addressed in this study. Except for the Xception & SVM and ResNet-18 & SVM classifiers, where the results for SEN and SPE are similar, other classifiers yielded SPE much lower than SEN. The differences are mainly due to the effect of unbalanced data, where the number of samples of < 5 years (*n* = 45) is less than those of > 5 years (*n* = 75). From the machine-learning standpoint, it is expected that the SPE rate can be improved if more biopsy samples can be obtained for enhancing the power of transfer learning from pretrained CNNs and SVM-based classification. Regarding the use of data augmentation, which is introduced in deep learning for creating more data by modifying existing samples, this study applied translation, rotation, and reflection to the training data but the classification performance was not better than without the use of data augmentation. Similar findings on the ineffectiveness of augmenting medical image data for transfer learning were reported in the literature [37, 38].

**Figure 2.**
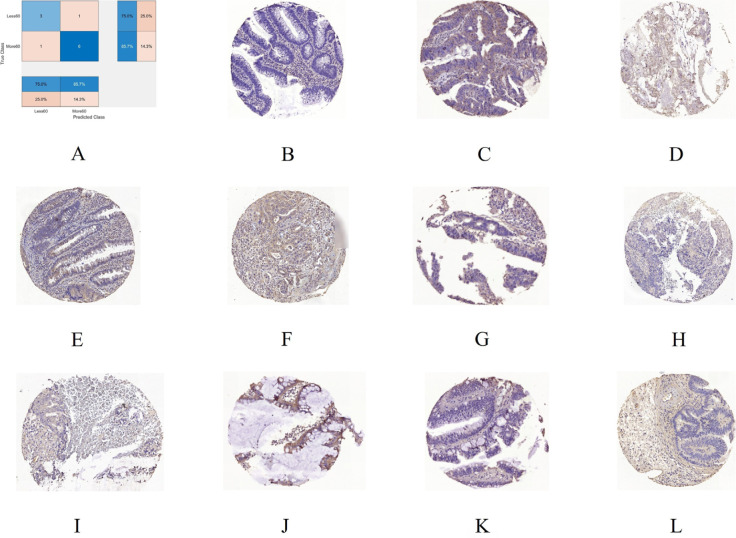
A 10-fold cross-validation using NASNet-Large & SVM. A. Confusion chart; B. an IHC sample of RhoB expression for < 5 years (60 months) misclassified as > 5 years; C. > 5 years misclassified as < 5 years; D, E, and F are 3 samples of correctly classified as < 5 years; G, H, I, J, K, and L are 6 samples correctly classified as > 5 years

**Figure 3.**
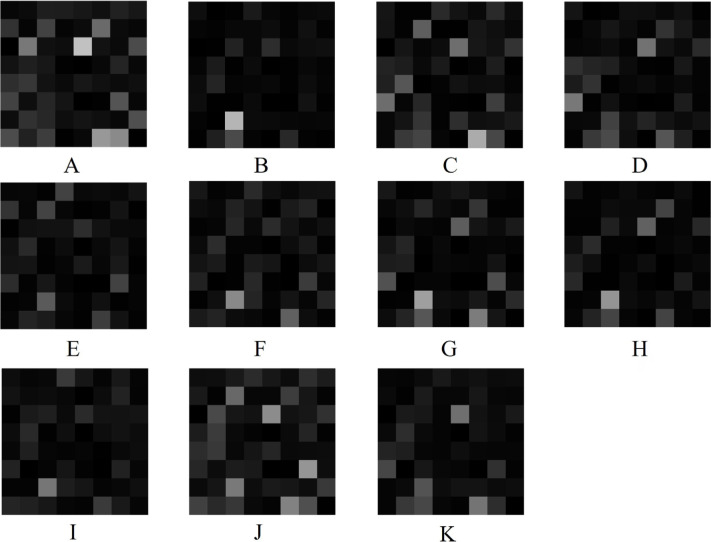
Extracted features at NASNet-Large layer “global_average_pooling2d_2” for IHC images of RhoB expression in rectal cancer biopsy. A, B, C, and D are deep features for < 5-year samples shown in Figure 2B, D, E, and F, respectively; E, F, G, H, I, J, and K are deep features for > 5-year samples shown in Figure 2C, G, H, I, J, K, and L, respectively

**Figure 4.**
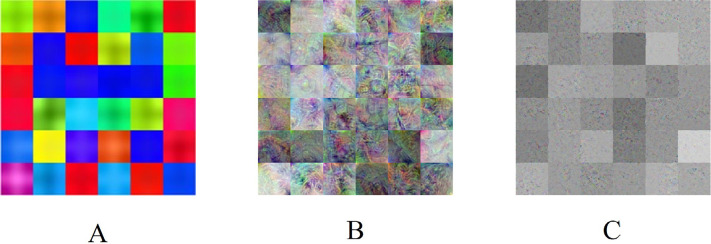
NASNet-Large features. A. Convolution layer “stem_conv1” (2nd layer), B. average pooling layer “normal_left3_8” (617th layer), and C. global average pooling layer “global_average_pooling2d_2” (1240th layer)

To gain insight into the deep-layer features of IHC images extracted from NASNet-Large, a 10-fold cross-validation was carried out in this study. The confusion matrixs shown in Figure 2 are the results provided by NASNet-Large & SVM and its associated IHC images. The features of these IHC images (Figure 3) extracted at the NASNet-Large layer of global average pooling “global_average_pooling2d_2” (layer number 1240), which is just before the fully connected layer of the network, can be visualized. It can be visualized that the feature images obtained from a much deeper layer as shown in Figure 3 are much more different than those of a low-level shown in Figure 4, where information about different colors and edges was captured. As in deeper layers, NASNet-Large learned to uncover finer-detail features of the input IHC images by further transforming the image properties extracted from earlier layers. Strong N (dark pixels), strong P (bright pixels), and weak (gray pixels) activations of the network transfer learning are shown in Figure 3. However, only the P activations were used because of the rectified linear unit (ReLU) that follows the “global_average_pooling2d_2” layer. The ReLU is a linear activation function defined as *f*(*x*) = max (0, *x*) which transforms the input as it is if the input is P; otherwise, it returns a zero if the input is N. Distinct patterns of P-activation patches between correctly classified images of < and > 5 years can be visualized (Figure 3B–3D *vs.* Figure 3F–3K).

Some observations about the activations of the feature maps as shown in Figure 3 can be elaborated as follows. Having mentioned previously, the network learns to detect complex features in deep convolutional layers and results in producing areas that are either bright (P) or dark (N). However, only the P activations are useful features for differentiating images of different classes. For the < 5-year class, the relatively most P (brightest) patches on the 8 × 8 grids are (3, 5) in Figure 3A, (7, 3) in Figure 3B, (8, 6) in Figure 3C, and (3, 5) and (6, 1) in Figure 3D. For the > 5-year class, the relatively brightest patches on the 8 × 8 grids are (7, 3) in Figure 3E, (7, 3) in Figure 3F, (7, 3) in Figure 3G, (7, 3) in Figure 3H, (7, 3) in Figure 3I, (3, 5) and (6, 7) in Figure 3J, and (3, 5) in Figure 3K. Although the spatial distribution of P activations in the IHC images of < 5 years are different from those of the ones of > 5 years, no consistency of locations of the relatively brightest patches can be found among the 4 IHC images of the < 5-year class. However, there is a consistency of the location of the relatively brightest patches among the 7 IHC images of the > 5-year class, which is a patch (7, 3), where patches (7, 3) in Figure 3I and 3K exhibit P activations to some degrees. Such a consistent P-activation pattern of the IHC images of > 5-year survival can be a useful factor for the relatively high performance of the NASNet-Large.

Because NASNet-Large could provide differentiable features of the two groups of the IHC images, the use of the SVM, which is well-known for its power of binary classification by being capable of separating data points into two classes, could therefore improve the classification performance over the direct use of the pretrained network for pattern classification. Regarding the computational time, NASNet-Large took about 500 min for data training with 20 epochs. The NASNet-Large & SVM-based classifier took less than 6 min for the network activations to extract the global average pooling features of the training IHC images and the SVM required only about 2 min for training the same data (total training time = 8 min). Both classification models were implemented on a single processor: Intel(R) Core(TM) i7-6500U CPU @ 2.50GHz. Such a time ratio of 500:8 illustrates an advantage of the NASNet-Large & SVM over the individual NASNet-Large in terms of computational speed by reducing the training time to about 63 times faster than the use of the single pretrained network. The time saving would become significantly higher for training a much larger dataset.

The dimensions of features extracted from the 6 pretrained CNN in ascending order are: 256 (AlexNet), 512 (ResNet-18), 1000 (SqueezeNet), 1920 (DenseNet-201), 2048 (Xeption), and 4032 (NASNet-Large). The classification ACC rates for CNN-SVM models in ascending order are: 67.28% (ResNet18-SVM), 67.28% (SqueezeNet-SVM), 69.09% (DenseNet201-SVM), 72.73% (AlexNet-SVM), 74.55% (Xception-SVM), and 84.55% (NASNet-Large-SVM). Although NASNet-Large-SVM and Xception-SVM, which have the largest feature dimensions, achieved higher ACC rates than the other four models, the whole comparison between feature dimensions and ACC rates does not indicate that the larger the dimensions are the higher the ACC is (AlexNet has the smallest number of feature dimensions but provided the third highest ACC).

Regarding the dimensions of the IHC samples, the largest, median, and smallest sizes of the images are about 3500 × 3300, 3200 × 3200, and 2700 × 2000 pixels, respectively, for each of the three red, green, blue (RGB) channels. By using the pretrained CNNs, all original images were resized to fit into the fixed input image size of each pretrained model as specified in Table 3. It is therefore not expected that different original image sizes would significantly affect the CNNs in learning the IHC expression of RhoB.

By definition, the standard deviation (*σ*) is calculated as

$$\sigma =\sqrt{\frac{\sum_{i}^{n}{\left(x_{i}-\bar{x}\right)}^{2}}{n-1}}$$

where *x_i_*, $\bar{x}$, and *n* are the value of sample *i*, sample mean, and the number of samples, respectively. In other words, standard deviation measures the dispersion of the samples relative to its mean. Hence it is not uncommon to have the SEN of NASNet-Large & SVM (90% + 11.76% > 100%). The problem is that the mathematical definition for *σ* does not consider an assumption of normality and the distribution of the SEN rates is heavily skewed [39, 40]. Here, *σ* indicates the square root of the average squared deviation, which is 11.76. If the standard error of the mean (*σ*_M_), which is defined as $\sigma_{M}=\sigma /\sqrt{n}$ [40], is used then $\sigma_{M}=11.76/\sqrt{5}=5.26$, giving the mean of SEN between 84.74% and 95.26%.

CNNs, whose architectures are analogous to that of the brain connectivity, learn to recognize images of different patterns by assigning learnable weights and biases to various aspects and details in the images. CNNs are also able to capture both spatial distribution and temporal information in images through the transformation of image kernels. Such artificial neural architectures can be trained to realize the complexity of images that are hidden from the human eye or too sophisticated to be revealed by conventional pattern analysis methods. Pathologists are used to investigating molecular characteristics in cancerous tissues stained by IHC for certain types of proteins and use statistical color descriptions and subjective experience to conclude the visual effects of the protein under study. In addition, manual analysis of RhoB expression in cancer biopsy is more limited than the analysis of tumor tissues obtained from surgical resection. Such limitation adds more challenge to the path of biomarker discovery. The use of AI for discovering RhoB as a potential biomarker captured on rectal-cancer biopsy illustrated in this study relieves the time-consuming, error-prone, and subjective task encountered by pathologists. Furthermore, results obtained from the proposed approach can be reproduced and improved when more relevant data become available.

Some limitations of the current study are as follows. This study used only the deep global average feature selected at the pooling layer before the fully connected layer of each pretrained CNN. Although the extraction of global average pooling features has certain advantages for SVM-based training and classification, exploration of features extracted at other deep layers and fusion of both shallow and deep features for classification are worth investigating. Another limitation, which is due to currently limited data for training AI models, is that the prediction of survival rates by means of the binary classification of either > or < 5 years does not consider the pTNM stage of patients with rectal cancer. The inclusion of cancer staging and other clinical and pathological characteristics together with their prior information into the IHC classification would be expected to improve the AI-based prediction and the outcome would be more clinically meaningful.

Radiomics [41, 42] is a relatively new concept in radiology that is mostly addressed in oncology. Radiomics aims to extract informative features or discover imaging biomarkers from tomographic data for the detection, diagnosis, and prognosis of cancer. As an extension, delta radiomics [43, 44] explores medical image features such as texture extracted from multiple image modalities at different time points of acquisition before and after chemotherapy and/or radiation therapy. In the case of rectal cancer, delta radiomics or delta texture analysis of magnetic resonance imaging (MRI) was carried out for predicting pathological complete responses and outcomes of patients who were treated with neoadjuvant chemoradiotherapy [45, 46]. Furthermore, texture analysis of computed tomography (CT) data for extracting radiomic features was found useful for differentiating malignant from benign lymph nodes in primary lung cancer [47, 48].

Texture analysis of radiology data such as MRI or CT in radiomics is based on grayscale levels of tissues. Features of IHC images extracted from pretrained deep-learning models such as the NASNet-Large used in this study are a kind of deep color texture generated mainly through a series of image convolution and transformation. It can be expected that these deep textural features can add a new dimension to the field of radiomics, where radiology data can be readily represented as input with pseudo color into a pretrained CNN model for mining deep textural features, to advance the state of the art of radiomics in cancer research.

In conclusion, based on the results presented and discussed in the foregoing sections, the integration of NASNet-Large and SVM appears a promising approach for validating RhoB as a biomarker expressed on the biopsy tissue of rectal-cancer patients. The ability to predict the cancer patient’s survival using biopsy is helpful because results given from the analysis of a biopsy can guide clinicians to determine the best course of action for the patient with a particular cancer type. Because the biopsy samples used in this study were taken from patients who were not treated with pRT or preoperative chemoradiotherapy (pCRT), assessment of tumor regression grading (TRG) was not clinically applicable. Evaluation of the impact of TRG is used for the management of rectal carcinoma patients treated with pCRT [49].

Not only the combined approach can provide high classification ACC, but also its data training requires much less computational time than the use of the transfer learning of a pretrained CNN for classifying IHC images. This advantage is particularly desirable for handling big pathological image data. It is because the SVM is trained with CNN-based features for image classification instead of retraining a pretrained deep-learning model for doing the same task, where the former strategy is much faster than the latter.

Furthermore, it can be expected that the classification ACC can be improved, particularly for the survival rate < 5 years (SPE), if more data and better data balance between the two survival rates become available as these two factors help build more reliable classification models through supervised learning with increased and balanced training data. This kind of real data balance may or may not be possible, depending on the on-going clinical collection of available data from future patients with rectal cancer. Otherwise, the use of artificial data augmentation is an alternative strategy for improving the AI-based prediction model.

The major limit of this research is the lack of external validation of the finding. We aim to externally validate the proposed prognostic model in a future study. External validation aims to provide evidence on the generalization of prognostic models, and there are several kinds of validation, depending on the hypothesis and patient size [50]. The biopsy samples used in this study were collected from the southeast health care region of Sweden, future patients’ data included in external validation are expected to be from different regions of Sweden or even countries. When new data become available, we may modify the AI approach, including parameter tuning, to make it more robust to be helpful for deeper clinical understanding and interventions. Furthermore, the main purpose of this study is to show that the predictive power of RhoB expression in rectal cancer biopsy can be discovered by the use of AI while our manual analysis could not reveal the prognostic capacity of the protein. Multivariate analysis of clinical parameters together with RhoB expression is worth conducting in our future study to strengthen evidence about the usefulness of this AI approach.

Being one of the leading causes of cancer-related death, the path to achieving PRE or personalized medicine for CRC is still far to travel because of the current lack of sufficient effective tools for early detection, prediction, prognosis, and treatment of the disease. It was reported that although the use of genetic profiles of CRC patients has been used for patient-specific treatment and measure of response to drugs, the clinical practice still suffers from the availability of a few CRC biomarkers [51]. Therefore, there is an urgent demand for discovering noninvasive or minimally invasive CRC biomarkers to pave the way for the development of effective new treatments for CRC and gain insights into the carcinogenesis of the disease [51].

## Abbreviations

ACC: accuracy
AI: artificial intelligence
CNNs: convolutional neural networks
CRC: colorectal cancer
DenseNet: dense convolutional network
FP: false positive
HE: hematoxylin and eosin
IHC: immunohistochemistry
N: negative
NASNet: neural search architecture network
P: positive
PRE: precision
pRT: preoperative radiotherapy
ResNet: residual network
RhoB: Ras homolog family member B
SEN: sensitivity
SPE: specificity
SVMs: support vector machines
TN: true negative
TP: true positive

## Declarations

### Availability of data and materials

The IHC image data used in this study are available at the first author’s homepage: https://sites.google.com/view/tuan-d-pham/codes under the name “Rectal cancer biopsy”.

### Copyright

© The Author(s) 2023.
